# Comparative genomics and genome biology of invasive *Campylobacter jejuni*

**DOI:** 10.1038/srep17300

**Published:** 2015-11-25

**Authors:** C. P. A. Skarp, O. Akinrinade, A. J. E. Nilsson, P. Ellström, S. Myllykangas, H. Rautelin

**Affiliations:** 1 Department of Medical Sciences, Clinical Microbiology, Uppsala University, Uppsala, Sweden; 2 Institute of Clinical Medicine, University of Helsinki, Helsinki, Finland; 3 Institute of Biomedicine, University of Helsinki, Helsinki, Finland; 4 Department of Bacteriology and Immunology, University of Helsinki, Helsinki, Finland

## Abstract

*Campylobacter jejuni* is a major pathogen in bacterial gastroenteritis worldwide and can cause bacteremia in severe cases. *C. jejuni* is highly structured into clonal lineages of which the ST677CC lineage has been overrepresented among *C. jejuni* isolates derived from blood. In this study, we characterized the genomes of 31 *C. jejuni* blood isolates and 24 faecal isolates belonging to ST677CC in order to study the genome biology related to *C. jejuni* invasiveness. We combined the genome analyses with phenotypical evidence on serum resistance which was associated with phase variation of *wcbK*; a GDP-mannose 4,6-dehydratase involved in capsular biosynthesis. We also describe the finding of a Type III restriction-modification system unique to the ST-794 sublineage. However, features previously considered to be related to pathogenesis of *C. jejuni* were either absent or disrupted among our strains. Our results refine the role of capsule features associated with invasive disease and accentuate the possibility of methylation and restriction enzymes in the potential of *C. jejuni* to establish invasive infections. Our findings underline the importance of studying clinically relevant well-characterized bacterial strains in order to understand pathogenesis mechanisms important in human infections.

*Campylobacter* is the most commonly reported cause of bacterial gastroenteritis, with an estimated financial burden of ~2.4 billion euros a year in the European Union (EU) alone^1^,^2^. Campylobacteriosis is usually a self-limiting disease, although in more severe cases hospitalization and antimicrobial treatment may be needed. Most infections are caused by *C. jejuni* and considered the main precedent for the development of Guillain-Barré syndrome (GBS); a severe demyelinating neuropathy, which is estimated to occur in 1/1.000-10.000 *Campylobacter* cases^3^,^4^. Bacteremia, another complication of *C. jejuni* infection with appreciable mortality rates^5^,^6^,^7^,^8^, has been estimated to occur in ~1% of *C. jejuni* infections^8^,^9^.

Multilocus sequence typing (MLST) is considered the golden standard for describing the population structure of *C. jejuni* and referral to *C. jejuni* as lineages such as sequence types (STs) and clonal complexes (CCs) is generally accepted as an informative tool^10^,^11^. MLST involves the analysis of seven housekeeping genes to deduce relationships between isolates and studies have shown that the *C. jejuni* population structure is weakly clonal^12^. Currently, more than 7.000 sequence types have been described (PubMLST database for *Campylobacter,* pubmlst.org/campylobacter, last accessed 22/04/2015).

The first whole genome sequence of *C. jejuni*^13^ revealed a small genome with a relatively low GC content (~1.6 Mpb; ~30.6% GC content), but known virulence factors, found in other intestinal pathogens, were not identified. Additional whole genome sequencing studies, including one or few *C. jejuni* strains, have demonstrated putative pathogenic factors to some extent^14^,^15^,^16^. However, the pathogenesis mechanisms of *C. jejuni* are still largely unknown. To date, the majority of the whole genome sequenced *C. jejuni* strains originate from human and animal faecal samples^13^,^14^,^16^. Also, *C. jejuni* strains from water and wildlife have been sequenced^15^. Although novel virulence mechanisms may be discovered when sequencing *C. jejuni* isolates derived from severe infections, like bacteremia, such studies are currently lacking.

We previously characterized blood and faecal *C. jejuni* isolates from Finnish patients and showed that MLST lineage ST677CC accounted for 48% of *C. jejuni* blood isolates^17^ and 11.7% of *C. jejuni* faecal isolates^18^. Moreover, this lineage has been only scarcely reported elsewhere in the world and makes up a bleak 0.2% of *C. jejuni* deposited in the pubMLST database (PubMLST database for *Campylobacter,* pubmlst.org/campylobacter, last accessed 22/04/2015). Interestingly, in our earlier study this particular lineage was declining among faecal isolates^18^ whereas it remained stable in numbers among the blood isolates, collected during the same time period as the faecal isolates^17^. A particular feature of the blood derived ST677CC isolates was their resistance to normal human serum (NHS). Although there was variation in the level of serum resistance, the average serum resistance of the ST677CC blood isolates was significantly higher than for those blood isolates belonging to other lineages^17^. Recently, Kivistö *et al.* (2014) described the comparative genomics of five ST677CC *C. jejuni* strains isolated at chicken farms and showed that the genomes of these strains were extremely homogeneous^16^. In the present study, we analyzed the genomes of 31 blood and 24 faecal *C. jejuni* isolates belonging to the ST677CC lineage to detect novel aspects of invasive *C. jejuni*. We also characterized the serum resistance of the faecal strains and combined the results with serum resistance data on the blood isolates^17^ and the genome data provided here.

## Results

### Phylogenomic overviews

**Figure 1:**
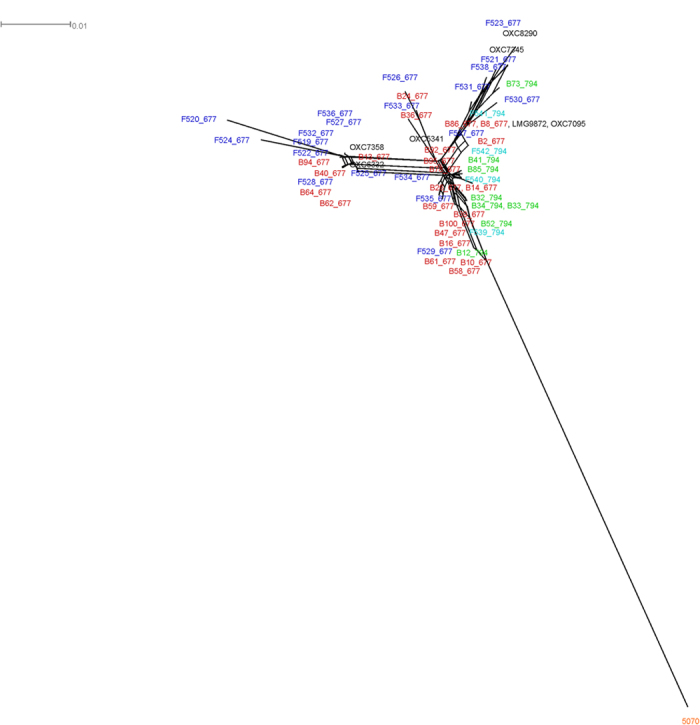
Phylogenomic overview of 63 *C. jejuni* strains belonging to the ST677CC lineage. For visualization purposes, the strains derived from blood have the prefix ‘B’ and the strains derived from faeces have the prefix ‘F’. Strains described in this study are shaded red (blood-derived ST677), green (blood-derived ST-794), blue (faecal ST-677) and teal (faecal ST-794). The farm-derived strain 5070 is shaded orange.

The genomes of the ST677CC strains were aligned with previously published *C. jejuni* genomes belonging to other lineages (Fig. S1). This analysis shows that the ST677CC lineage is rather unique and clusters away from other commonly found lineages ST21CC and ST45CC. A second whole genome alignment of the ST677CC strains only (n = 63, including the 55 strains characterized in this study) revealed that the two sublineages, ST-677 and ST-794, showed different distribution patterns; ST-677 was not confined to a single cluster, whereas ST-794 was mainly found in one cluster together with several ST-677 strains (Fig. 1). The clinical strains did not cluster according to the source of origin but rather, smaller clusters containing strains derived from both blood and faeces were found (Fig. 1). However, the only non-clinical strain 5070^16^, clustered away from the clinical strains.

A second approach was used to refine the ST677CC genome comparisons by using the core genome alignment tool Parsnp (http://harvest.readthedocs.org/en/latest/content/parsnp.html, last accessed 22/04/2015). In this analysis ST-677 and ST-794 were well separated from one another (Fig. S2) and the chicken farm-derived strain (5070) clustered together with the ST-677 strains.

### Association of serotype, capsular features and serum resistance

All ST677CC strains in our study belonged to heat stable capsule type HS4^19^, but the HS4 capsule of the ST677CC strains could be further subdivided into four different groups (Fig. S3). Group A contained the majority of the ST677CC strains (39/55) and possessed another gene cluster located between HS4.07 and HS4.08. The second HS4 CPS group (B) was similar to group A, except that group B had an inverted HS4.07 located outside the capsule locus. This group contained only six strains, all derived from blood. The third group (C) also contained six strains and was similar to group A, except that the region spanning HS4.08-HS4.15 was inverted and the extra gene cluster was located between HS4.15 and HS4.16 (Fig. S3A). Finally, the last group (D) included four strains and was divided into two clusters, separated by a long stretch of unrelated DNA (Fig. S3B). Also, all four groups carried a *C. jejuni* subsp. *doylei* like capsular gene cluster earlier described by Kivistö *et al.* (2014)^16^. Common to all ST677CC strains was the loss of the upstream homopolymeric G tract and subsequent disruption of the HS4.07 ORF. The *C. jejuni* capsule has been associated with serum resistance^20^ and earlier we have observed that the ST677CC strains from blood exhibit higher levels of serum resistance than those strains belonging to other lineages^17^. Here, we performed serum sensitivity assays on 31 faecal ST677CC isolates, of which 23 were also genome sequenced in this study. The faecal ST677CC isolates exhibited a wide range of serum sensitivity, with an average serum resistance of 45%, which is comparable to the earlier observed serum resistance of the blood isolates^17^. Subsequently, we looked for features in the capsule loci of ST677CC that may attribute to the observed levels of serum resistance. We found that the homopolymeric tract length of *wcbK*, encoding a GDP-mannose 4,6-dehydratase could be associated to different levels of serum resistance. Two different groups of strains were observed; the first group contained a *wcbK* with a 9-G homopolymeric tract and was predicted to encode an intact *wcbK* and the second group, with 8-G and 10-G homopolymeric tracts, putatively encoded a disrupted *wcbK* (Table 1). The average serum resistance of strains belonging to the first group (containing an intact *wcbK* ORF with a 9-G homopolymeric tract) was significantly lower than that of the strains belonging to the second group (Table 2).

### Restriction modification systems

**Figure 2:**
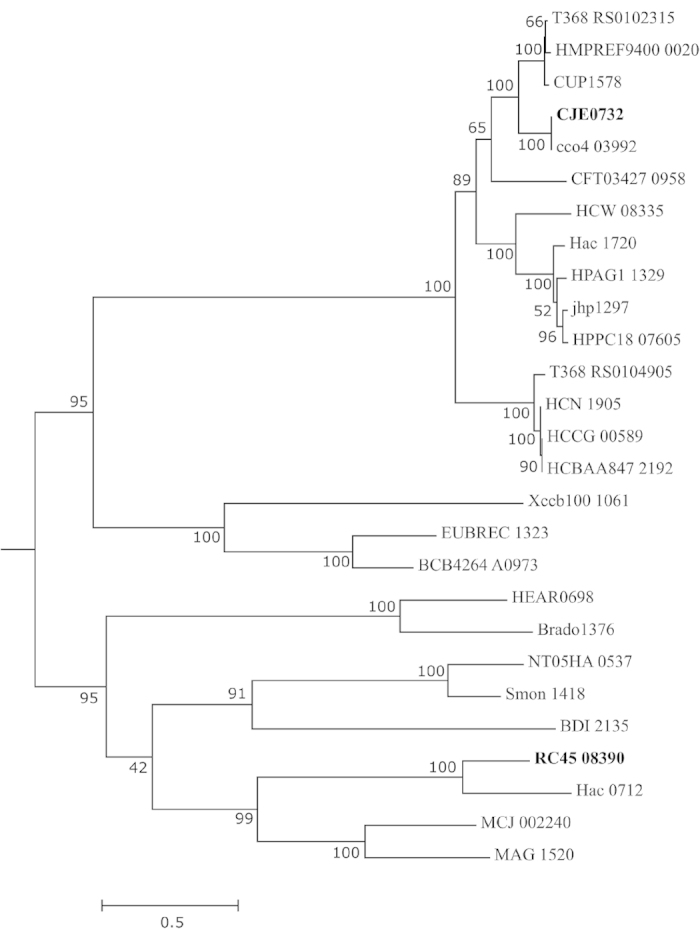
Phylogeny of Type III restriction modification system protein sequences. The Type III RM of ε-proteobacteria *C. jejuni* RM1221 (bold-faced) and *C. jejuni* 539 (ST-794; bold-faced), *C. coli, C. upsaliensis* JV21, *Helicobacter acinonychis and Helicobacter bilis* are included and related sequences of organisms are listed in Table S1. Amino acid sequences were aligned using MAFFT (http://mafft.cbrc.jp/alignment/server/index.html), last accessed 22/04/2015 and cleaned up using Gblocks^56^. Maximum likelihood analysis was performed in MEGA6^54^ as described in Materials and Methods.

We describe a novel Type III restriction modification (RM) system among ST677CC sublineage ST-794. A blastP query with the restriction subunit against the Concise Microbial Protein Database, http://www.ncbi.nlm.nih.gov/genomes/prokhits.cgi,last accessed 22/04/2015 showed ten homologs in distant species and only one in ε-proteobacteria: *Helicobacter acinonychis* (*Hac*) (Table S2). Phylogenetic reconstruction of the restriction (res) subunit tCDS (RC45_08390; strain 539) showed that the res subunit of ST-794 was phylogenetically distinct from those found in other ε-proteobacterial species (Fig. 2). Inspection of the genomic region with RAST’s integrated SEED viewer focusing on the res subunit showed that it was similar to res subunits found among several *Mycoplasma* species (Fig. S4). The Type III RM was in a cluster together with several hypothetical proteins and an integrase and was located next to the tRNA-Val.

BlastP queries on our dataset of an earlier identified *Helicobacter pylori*-like Type II RM^16^, consisting of ulcer-associated endonuclease *iceA1* and ulcer-associated adenine-specific DNA methyltransferase *hpyIM* and an additional orphan DNA methylase showed that this particular cluster was also present among all our 55 ST677CC strains.

Two disrupted Type I RM systems, one disrupted Type IV (McrBC system) and a unique adenine-specific DNA methylase were found among our 55 strains.

### *Campylobacter jejuni* integrated elements (CJIE)

**Figure 3:**
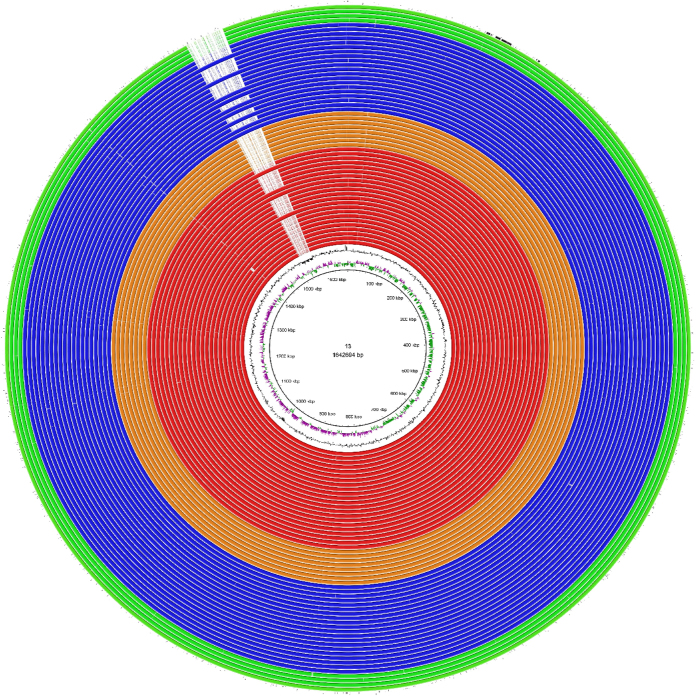
BRIG representation of CJIE5 among ST677CC *C. jejuni* blood and faecal strains. Blood strain 13 was used as a reference. Blood strains belonging to ST-677 are colored red, blood strains belonging to ST-794 are colored orange. Faecal strains belonging to ST-677 are colored blue, faecal strains belonging to ST-794 are colored green. RM1221-CJIE1 is plotted in black on the outer circle.

Currently, four CJIE are known for *C. jejuni* of which CJIE1 and CJIE2 were detected among our strains (Table 1). Although CJIE1 was present among all our strains, 16 strains contained CJIE1 which missed 24 ORFs, most of which correspond to phage-related and hypothetical proteins. CJIE2 was only present among 11 strains; six derived from blood and five derived from faeces (Table 1). CJIE3 and CJIE4 were not found among our strains. We present here a putative fifth *C. jejuni* integrated element (CJIE5), which was found among 14 ST-677 strains only (Table 1, Fig. 3). This element spans a ~28 kb region and was located next to tRNA-Leu. Two endonucleases were identified on CJIE5: a DNA/RNA non-specific endonuclease (red ORF in Fig. S5) and an Eco57I endonuclease (green ORF in Fig. S5).

### Virulence traits

Previously identified virulence trait cytolethal distending toxin (CDT)^21^ was disrupted among our ST677CC strains. Also, other virulence traits such as genes for lipooligosaccharide sialylation, and metabolism related virulence factors γ-glutamyl transpeptidase (GGT) and fucose permease (Cj0486) were all absent from the ST677CC lineage. Conversely, iron acquisition of the ST677CC strains resembled that of the highly virulent strain 81–176^16^,^22^. Three putative Type Vb secretion systems (T5bSS), two of which cluster with a filamentous haemagglutinin domain protein (FHA) were recently identified among the ST677CC lineage^16^ and were also present among our 55 ST677CC strains (data not shown).

## Discussion

Generally, the ST677CC genomes characterized in this study were similar in size (Table 1) and GC content (~30.3%) as previously shown for *C. jejuni* genomes^13^,^14^. In the phylogenomic overview, including strains belonging to other lineages, the ST677CC strains formed a tight cluster away from other major lineages, such as ST21CC and ST45CC. In a more detailed whole genome analysis only including ST677CC strains, smaller clusters for ST-677 were observed and one major cluster for ST-794 was found. However, some ST-677 and ST-794 clustered closely with one another and a further analysis, only including the core genomes of the ST677CC strains, indicated that the similarities between the two STs in the whole genome analysis could be mainly due to a partially shared pool of accessory gene content. Accessory gene content may be acquired in a host-dependent manner, which is supported by the whole genome analysis in which the chicken farm strain clustered far away from the clinical strains. Once available, it will be interesting to perform analyses with ST677CC strains derived from a wider range of hosts and environments and attempt to detect traces of host-dependent horizontal gene transfer (HGT).

One of our earlier major findings among the blood isolates belonging to the ST677CC lineage was the overall higher serum resistance when compared to other major *C. jejuni* lineages ST21CC and ST45CC^17^. Here, we performed additional serum resistance studies with the faecal ST677CC isolates, which showed that these strains exhibited a wide range of serum sensitivity with an average serum resistance comparable to that of the blood isolates^17^. These results indicate that the ST677CC lineage is intrinsically more serum resistant than other major *C. jejuni* lineages and that serum resistance is not dependent on the site of isolation.

The capsule of *C. jejuni* has been implied to play an important role in serum resistance and the development of *C. jejuni* mediated bacteremia^20^. All ST677CC strains in our study belonged to capsule type HS4, which has commonly been detected among *C. jejuni* derived from blood cultures^8^. Interestingly, we found four different HS4 capsule groups pronounced by genomic rearrangements, which occurred adjacent to the open reading frame (ORF) of disrupted HS4.07 (encoding an *O*-methyl phosphoramidate (MeOPN) transferase). It is likely that the observed truncation and loss of the homopolymeric G tract upstream of this ORF are due to these frequent recombinational events.

We further looked into a proposed association of phase-variated *wcbK* and serum resistance^16^. We found that *C. jejuni* strains which have disrupted *wcbK* exhibited a higher serum resistance than those strains encoding an intact *wcbK.* Interestingly, these results are in line with the skewed distribution of *C. fetus* subsp. *fetus* serotype A which is more commonly found among blood isolates than serotype B. This phenomenon has been attributed to the absence of *wcbK* in serotype A and moreover the increased serum resistance of serotype B Δ*wcbK* mutants^23^. Thus, it is feasible that absence of the GDP-mannose 4,6-dehydratase product on the HS4 capsule most likely led to higher serum resistance, possibly leading to prolonged survival in the bloodstream and subsequently the establishment of bacteremia. These results indicate that certain *C. jejuni* capsular structures more closely resemble those of *C. fetus* subsp. *fetus* and that functions of capsular structures can cross the species boundaries in *Campylobacter*.

*C. jejuni* is considered a naturally competent organism and contains horizontally acquired gene content^24^,^25^,^26^. Nevertheless, restriction modification systems, orphan methylases and endonucleases are ubiquitous among *C. jejuni*^14^,^25^,^26^. We describe a unique Type III RM system among strains belonging to sublineage ST-794 and show that it is distinct from previously characterized Type III RM systems in *Campylobacter* and *H. pylori*. It is likely that this Type III RM has been horizontally acquired from a distant species, possibly as part of a small conjugative element. Another putatively horizontally acquired RM system was found among ST677CC strains (this study and 16) and consists of an ulcer-associated endonuclease, similar to *H. pylori iceA1* and ulcer-associated adenine-specific DNA methyltransferase *hpyIM.* Homologs of these proteins are found in *Riemerella anatipestifer* and *Gallibacterium anatis* (previously *Pasteurella anatis*); pathogens responsible for causing bacteremia in ducks and laying hens, respectively, but these homologs are absent among *C. jejuni* belonging to other major lineages^16^. Moreover, two orphan adenine-specific methylases were also identified, one of which was located in the same cluster as the *iceA1*/*hpyIM* genes. Recently, unique adenine methylation profiles of a sheep abortion clone were found when compared with methylation profiles of enteritis isolates^27^ and adenine methylation has been shown to play a vital role in the establishment of *Salmonella* Typhimurium in deeper tissue sites^28^. These observations indicate that methylation could play a substantial role in the establishment of invasive infections and both the ST-794 Type III RM and the *iceA1/hpyIM* gene cluster could represent additional methylation mechanisms in *C. jejuni* which will require further characterization.

Degeneration of RM systems may be associated with the presence of mobile genetic elements^29^,^30^. Four *C. jejuni* integrated elements (CJIE1-4) have been identified so far; of which CJIE1 has been the one most commonly found and best characterized^31^ and was also found among all our strains. Here, we found a fifth and novel integrated element, CJIE5 among a subset of ST-677 strains, which encodes two additional restriction endonucleases. Interestingly, the presence of CJIE5 seemed to coincide with the observed loss of CJIE1 ORFs corresponding to prophage I and F proteins, tail fiber protein H, baseplate assembly proteins J and V and several hypothetical proteins (Table 1). The absence of these ORFs was more commonly observed among our faecal strains than our blood strains. The driving force behind this jump-out is elusive; however as it is likely that our blood-derived *C. jejuni* first travelled through the gut, it is possible that selection for the absent ORFs may have occurred during gut passage, a phenomenon earlier demonstrated in chickens^32^. Four of the missing ORFs have been shown to be induced upon exposure to bile acid^33^ and collectively, these observations suggest that resistance to bile may play a role in disrupting CJIE1.

Integrated element CJIE3 was not found in the ST677CC lineage. This is interesting as presence of a Type VI secretion system on CJIE3 was shown earlier^34^,^35^, and was involved in lysing erythrocytes and suggested to be important in the establishment of invasive infections^35^. However, its absence among the ST677CC lineage indicates that its presence is not required for invasive infection by the ST677CC lineage. Additionally, the traditional virulence trait cytolethal distending toxin^21^ was found to be disrupted among all the ST677CC strains (this study and 16). Further virulence traits such as genes for lipooligosaccharide sialylation, and metabolism related virulence factors γ-glutamyl transpeptidase (GGT) and fucose permease (Cj0486) were all absent from the ST677CC lineage, which is in agreement with earlier locus-based presence/absence PCR screening studies^36^,^37^. On the other hand, iron acquisition of the ST677CC strains has been shown to resemble that of the highly virulent strain 81–176^16^,^22^. Interestingly, three putative Type Vb secretion systems (T5bSS) were recently identified among the ST677CC lineage^16^ and were also present among our 55 ST677CC strains (data not shown). Two of the putative T5bSS clustered with a filamentous haemagglutinin domain protein (FHA)^16^, a paralog of which has earlier demonstrated adhesive abilities^38^. These systems, yet especially the two distinct FHA proteins, could represent novel invasion proteins among *C. jejuni*.

Our study shows that the ST677CC lineage has the potential to encode a diverse arsenal of epigenetic regulatory mechanisms which could have played a role in the invasive nature of the strains. However, absence or truncation of previously defined virulence factors indicate that such are not necessarily detected among invasive isolates. This makes it increasingly clear that previously adopted ‘virulence genes’, described for a limited number of strains, may not be widely distributed among the species. Our study highlights the importance of global genomic and phenotypical characterization of a representative number of strains associated with particular clinical outcomes, which deserves consideration in the design of future molecular microbiological studies.

## Materials and Methods

### Bacterial isolates and DNA extraction

The bacterial isolates characterized in this study were derived from human bacteremia or gastrointestinal infections. Frozen stocks of a total of 31 blood isolates^9^ and 31 faecal isolates^18^,^39^ were cultivated onto Columbia blood agar (CBA; Oxoid, Basingstoke, UK) plates and incubated at 42 °C for 24–48 hours in a microaerobic atmosphere (CampyGen; Oxoid). DNA was extracted with the Qiagen DNeasy Blood and Tissue kit (Qiagen Sciences, Germantown, MD, USA), and subjected to whole genome sequencing.

### Whole genome sequencing, assembly and visualization

Whole genome sequencing was conducted using the MiSeq Desktop Sequencer (Illumina, San Diego, CA, USA). For each sample, 50 ng of extracted DNA was applied for sequencing library construction using the Nextera XT DNA Sample Preparation and Indexing Kits (Illumina). Sample preparation and 24-plex indexing were performed according to the manufacturer’s instructions. Normalisation of the quantities of pooled samples was done by measuring the DNA concentrations of individual sequencing libraries using Qubit^®^ (Life Technologies, Carlsbad, CA, USA). Paired-end sequencing (2×251 bases) with two index reads was performed using the MiSeq Reagent Kit v2 (Illumina) at Blueprint Genetics Oy, University of Helsinki.

Quality of raw Illumina paired-end reads was checked using FastQC 40. The reads were trimmed using trimomantics in paired-end mode^41^. *De novo* assembly of the reads was done using SPAdes 3.0.0 and a5 miseq pipeline (20140113)^42^,^43^. In addition to the assemblers’ built-in scaffolder, SSPACE v3^44^, a standalone scaffolder, was used to generate scaffolds from assembly contigs. Gaps within the scaffolds were closed using GapCloser v1.12^45^. In each stage, results generated by different tools were compared in order to choose the optimum assembly using QUAST v2.3^46^. Mauve^47^ was used to re-order the supercontigs using *C. jejuni* strains RM1221 and ATCC 43432 as reference genomes (accession numbers: NC_003912 and HQ343267). Primary annotation of all strains was performed using Rapid Annotation using Subsystem Technology (RAST)^48^, and later, the sequences were manually curated using Artemis^49^. Final annotation was done using NCBI Prokaryotic Genome Annotation Pipeline (NCBI_PGAP). All analyses were conducted in the high-performance supercluster Taito at CSC (IT-Center of Science), Finland.

BLAST Ring Image Generator (BRIG) was used to visualize genome comparisons^50^.

### Phylogenomics

The fragmented alignment tool Gegenees^51^ was used to perform two reciprocal BLAST-assisted whole genome alignments to obtain phylogenomic overviews. First, our 55 ST677CC genomes were aligned with 51 previously sequenced *C. jejuni* strains which belonged to 16 different lineages to obtain a phylogenomic overview of the species. For this alignment, blastN was performed with 500 bp fragment sizes and a sliding window (step size) of 500 bp. Secondly, our ST677CC genomes together with the genomes of six ST677CC strains from human gastroenteritis in England (the Oxford sentinel surveillance study; http://pubmlst.org/campylobacter/info/Oxfordshire_sentinel_surveillance.shtml, last accessed 22/04/2015), one ST677CC strain from a meningitis case in Sweden and the recently described chicken farm-derived ST677CC strain 5070^16^. The BlastN option was used with 200 bp fragment sizes and a sliding window of 100 bp. Threshold levels were set at 0% of the maximum score values. The pair-wise similarities were exported in Nexus file format and visualized in SplitsTree4^52^ as a split network. To visualize the core genome of the ST677CC lineage only, the harvest suite tool Parsnp (http://harvest.readthedocs.org/en/latest/content/parsnp.html), last accessed 22/04/2015 was used^53^. A randomly chosen reference was aligned against all ST677CC genomes (-c) using recombination detection (-x). The tree was visualized using MEGA6^54^.

### Serum sensitivity assay and serotyping

Serum sensitivity assays were performed as described previously^17^. Serotyping was performed using commercially available serotyping set (Campylobacter Antisera Seiken Set, Denka Seiken, Tokyo, Japan) based on heat-stable Penner’s antigen as previously described^55^. Antigens from *Campylobacter* were extracted with nitric acid and sensitized to chicken erythrocytes. They were then tested against 25 different antisera and checked for agglutination according to the manufacturer’s instructions.

### Phylogenetic analysis

For reconstruction of the phylogeny of the res subunit of the ST-794 Type III RM system the amino acid sequences of ε-proteobacterial Type III res subunits and other Type III res subunits were aligned using MAFFT L-Ins-i at http://mafft.cbrc.jp/alignment/server/index.html, last accessed on 22/04/2015, and cleaned up using Gblocks^56^. The phylogeny was reconstructed by maximum likelihood analysis in MEGA6^54^ with 500 bootstraps using the WAG substitution model, G+I rates, SPR heuristics and made use of all informative sites left after Gblocks cleaning.

## Additional Information

**How to cite this article**: Skarp, C. P. A. *et al.* Comparative genomics and genome biology of invasive *Campylobacter jejuni*. *Sci. Rep.* **5**, 17300; doi: 10.1038/srep17300 (2015).

## Supplementary Material

Supplementary Information

## Figures and Tables

**Table 1:** Main characteristics of ST677CC *C. jejuni* genomes of blood isolates (numbered 2–100) and faecal isolates (numbered 519–542).

Strain	ST	Genome size (Mbp)	No. of contigs†	CJIE1	CJIE2	CJIE5**	Type III RM***	HS4 CPS group	*wcbK*hp G-tract
2	677	1.67	26	√	√			B	10
8	677	1.62	28	√				A	10
10	677	1.65	26	√*	√			B	**9**
13	677	1.64	18	√*		√		B	10
14	677	1.67	22	√	√			A	10
16	677	1.64	21	√				A	**9**
24	677	1.66	22	√				B	**9**
26	677	1.63	23	√				B	**9**
36	677	1.66	29	√				A	**9**
39	677	1.63	17	√				C	**9**
40	677	1.64	28	√*		√		A	**9**
47	677	1.63	17	√				A	**9**
58	677	1.63	19	√				A	10
59	677	1.63	19	√				A	10
61	677	1.63	17	√				A	**9**
62	677	1.64	19	√*		√		A	10
64	677	1.64	18	√*		√		A	10
78	677	1.63	27	√				A	**9**
86	677	1.63	18	√				A	**9**
92	677	1.63	17	√				A	**9**
94	677	1.64	21	√*		√		A	**9**
95	677	1.63	36	√				A	10
100	677	1.67	17	√	√			A	10
12	794	1.63	15	√			√	A	10
32	794	1.67	21	√	√		√	A	**9**
33	794	1.63	14	√			√	D	**9**
34	794	1.63	18	√			√	A	10
41	794	1.63	17	√			√	A	**9**
52	794	1.63	17	√			√	B	**9**
73	794	1.67	16	√	√		√	A	**9**
85	794	1.63	19	√			√	A	**9**
519	677	1.64	21	√*		√		C	**9**
520	677	1.68	28	√*		√		A	**9**
521	677	1.67	16	√	√			C	10
522	677	1.64	19	√*		√		A	10
523	677	1.66	19	√	√			A	**9**
524	677	1.67	49	√*		√		D	10
525	677	1.64	15	√*		√		A	**9**
526	677	1.66	20	√				A	**9**
527	677	1.64	20	√*		√		C	10
528	677	1.64	16	√*		√		A	10
529	677	1.63	27	√				A	**9**
530	677	1.65	28	√*	√			A	**9**
531	677	1.66	19	√	√			A	10
532	677	1.64	19	√*		√		A	10
533	677	1.65	37	√				A	**9**
534	677	1.63	15	√				C	**9**
535	677	1.63	18	√				D	**9**
536	677	1.64	15	√*		√		C	**9**
537	677	1.63	20	√				A	**9**
538	677	1.67	18	√	√			A	10
539	794	1.63	16	√			√	A	10
540	794	1.63	16	√			√	A	8
541	794	1.63	20	√			√	A	**9**
542	794	1.63	13	√			√	D	10

Strains are ordered by origin (blood and faeces) and sequence type (ST-677 and ST-794). The ST-794 strains are shaded grey. Presence and absence of *C. jejuni* integrated elements (CJIE1, CJIE2 and CJIE5), HS4 capsule (CPS) subdivisions and the *wcbK* homopolymeric (hp) G-tract is shown. Intact *wcbK* ORFs are indicated in bold in the last column.

^†^Number of contigs before *in silico* gap closing, concatenation and re-ordering of the supercontigs.

^*^Partial CJIE1 sequence.

^**^CJIE5: Newly identified in this study.

^***^Type III restriction-modification (RM) system which was unique to the ST-794 sublineage.

**Table 2:** Association of *wcbK* homopolymeric G tract with serum resistance in the ST677CC lineage.

No. of G’s	No. of blood strains	No. of faecal strains*	Avg. serum resistance (%)	p-value
8**	0	1	62	
10**	12	9		
9***	19	13	33.5	<0.01†

^*^For one faecal strain serum resistance was not performed.

^**^wcbK gene ORF truncated.

^***^wcbK gene ORF intact.

^†^Student’s t-test (intact *wcbK* ORF with 9 G’s vs truncated *wcbK* ORF with 8 or 10 G’s).
