# Supplementary material for: Comparative genomics and genome biology of invasive Campylobacter jejuni
Source: Sci Rep. 2015 Nov 25;5:17300. doi: 10.1038/srep17300 (PMC4658567; doi:10.1038/srep17300)
Supplement: Supplementary Information [file srep17300-s1.pdf]

Supplementary material ‘**Comparative genomics and genome biology of invasive *Campylobacter jejuni*’**

C.P.A. Skarp

O. Akinrinade

A.J.E. Nilsson

P. Ellström

S. Myllykangas

H. Rautelin

## Legends

Figure S1. Phylogenomic overview of 106 *C. jejuni* strains, including 55 *C. jejuni* strains belonging to the ST677CC lineage described in this study. Strains belonging to ST21CC (15 strains) are shaded green with reference strain 11168 bold-faced. Strains belonging to ST45CC (three strains) and ST283CC (strain 81116 only) are shaded red with reference strain 4031 bold-faced. The ST677CC strains (63 strains) are shaded blue and two strains (faecal strains 524 and 542) representing the two major STs (ST-677 and ST-794) belonging to this lineage are bold-faced. Due to the homogeneity in large parts of ST677CC, the majority of labels were removed to enhance clarity of the figure.

Five strains (255, 414, 1336, 30318 and K1) did not belong to any lineage and are black and bold-faced. The remaining strains belonged to ten different lineages, which are shaded as follows: ST22CC, orange (strains BJ-CJD101 and HB93-13); ST42CC, light green (strain 81-176); ST52CC, pink (strains 10186, 20176 and CG8421); ST353CC (strain R14) and ST354CC (strains RM1221 and S3), purple; ST362CC, teal (strains 260.94, HN-CJD07035, ICDCCJ07001 and ICDCCJ07002); ST403CC (strain ATCC33560) and ST460CC (strain

10227), light red; ST464CC, light green (strain Cj5) and ST574CC, yellow (strains 30286, CG8486 and Cj3).

Figure S2. Core genome analysis of 63 *C. jejuni* ST677CC strains. Number 1 represents the ST-794 strains; number 2 represents the ST-677 strains and includes one strain (OXC8290) typed as ST-6514, shaded purple. The Harvest suite tool Parsnp (http://harvest.readthedocs.org/en/latest/content/parsnp.html, last accessed on 22/04/2015) was used for core genome determination.

Figure S3. HS4 capsule locus of 55 *C. jejuni* strains belonging to the ST677CC lineage. (a) HS4 groups A-C. (b) HS4 group D.

Figure S4. Similarity of *C. jejuni* ST-794 Type III restriction modification system and *Mycoplasma* Type III RM. Number one (red arrow) represents the restriction subunit (2019 bp; 673 aa); number two (green arrow) represents the methylation subunit (1611 bp; 537 aa).

Figure S5. Artemis view of CJIE5 in ST-677 blood isolate 40. ORFs marked in yellow are considered to belong to CJIE5. The ORF marked in red represents a DNA/RNA non-specific endonuclease. Interproscan of the ORF marked in green found an adenine-specific DNA methylase signature, a Type III res subunit, and a DEAD-like helicase superfamily C-terminal domain.

Table S1. List of species included in our ε-proteobacterial database for orthology querying.

Table S2. BlastP results of the ST-794 Type III res subunit against the Concise Microbial

Protein Database

Table S3. Accession numbers of the whole genome sequences of 55 *C. jejuni* strains isolated from human blood (n=31; strains 2-100) and faeces (n=24; strains 519-542).

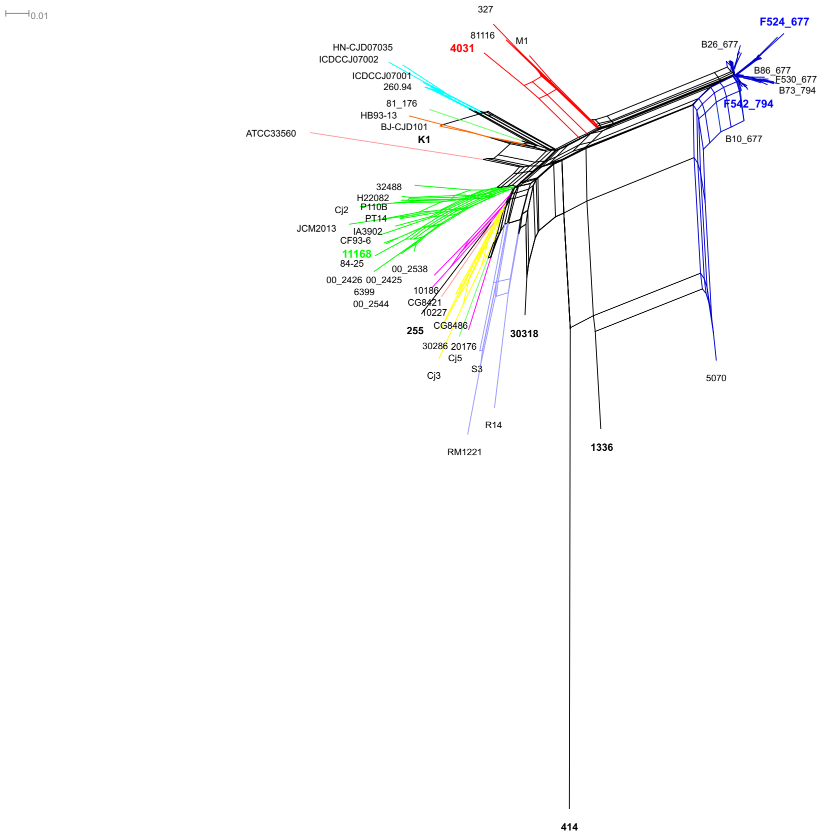

0.01
327
81116
M1
4031
HN-CJD07035
ICDCCJ07002
ICDCCJ07001
260.94
81_176
HB93-13
BJ-CJD101
K1
ATCC33560
F524_677
B26_677
B86_677
F530_677
B73_794
F542_794
B10_677
32488
H22082
Cj2
P110B
PT14
JCM2013
IA3902
CF93-6
11168
84-25
00_2538
00_2426
00_2425
6399
00_2544
10186
CG8421
10227
CG8486
255
30318
30286
20176
Cj5
S3
Cj3
R14
RM1221
1336
5070
414

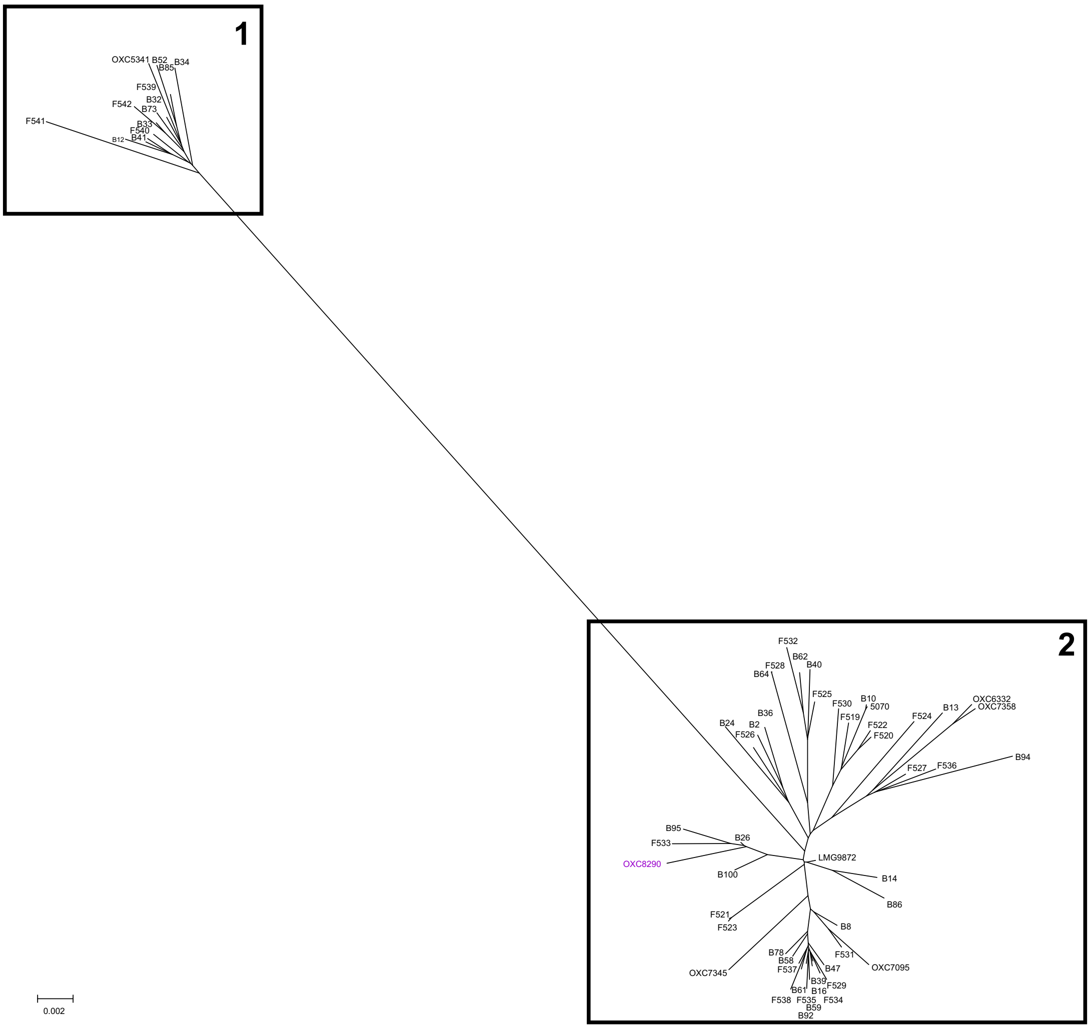

1
OXC5341
B52
B34
B85
F539
B32
F542
B73
F541
B33
F540
B12
B41
2
F532
B62
F528
B40
B64
F525
F530
B10
5070
B36
F519
F524
B13
OXC6332
OXC7358
B24
B2
F522
F526
F520
B94
F527
F536
B95
F533
B26
OXC8290
LMG9872
B100
B14
B86
F521
F523
B8
B78
F531
B58
OXC7095
F537
B47
OXC7345
B39
F529
B61
B16
F538
F535
F534
B59
B92
0.002

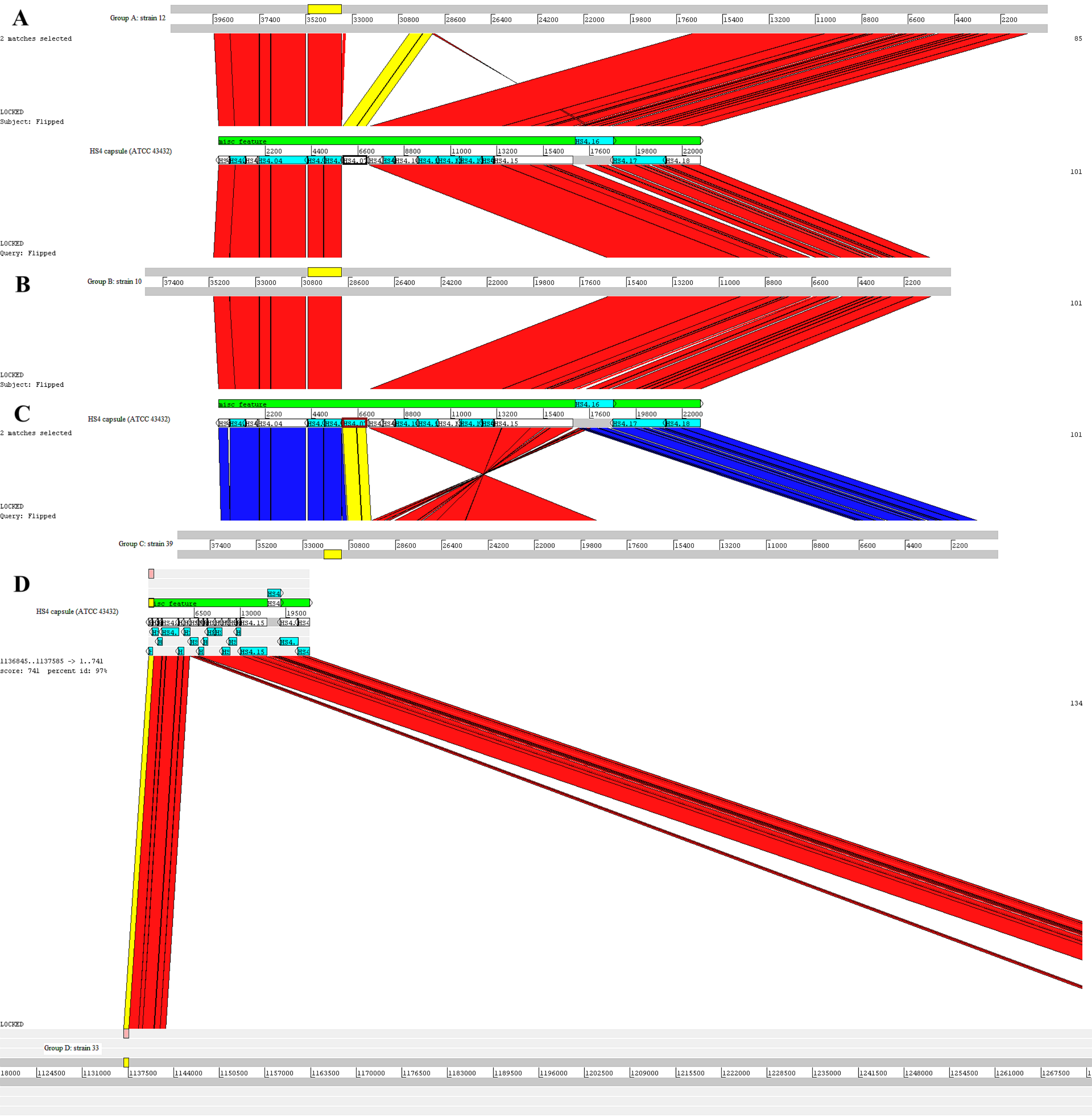

A
Group A: strain 12
39600 37400 35200 33000 30800 28600 26400 24200 22000 19800 17600 15400 13200 11000 8800 6600 4400 2200
2 matches selected
85
LOCKED
Subject: Flipped
misc feature
HS4.16
HS4 capsule (ATCC 43432)
2200 4400 6600 8800 11000 13200 15400 17600 19800 22000
HS4.04
HS4.15
HS4.17
HS4.18
101
LOCKED
Query: Flipped
B
Group B: strain 10
37400 35200 33000 30800 28600 26400 24200 22000 19800 17600 15400 13200 11000 8800 6600 4400 2200
101
LOCKED
Subject: Flipped
C
misc feature
HS4.16
HS4 capsule (ATCC 43432)
2200 4400 6600 8800 11000 13200 15400 17600 19800 22000
HS4.04
HS4.15
HS4.17
HS4.18
2 matches selected
101
LOCKED
Query: Flipped
Group C: strain 39
37400 35200 33000 30800 28600 26400 24200 22000 19800 17600 15400 13200 11000 8800 6600 4400 2200
D
HS4 capsule (ATCC 43432)
misc feature
6500 13000 19500
HS4.15
1136845..1137585 -> 1..741
score: 741 percent id: 97%
134
LOCKED
Group D: strain 33
18000 1124500 1131000 1137500 1144000 1150500 1157000 1163500 1170000 1176500 1183000 1189500 1196000 1202500 1209000 1215500 1222000 1228500 1235000 1241500 1248000 1254500 1261000 1267500

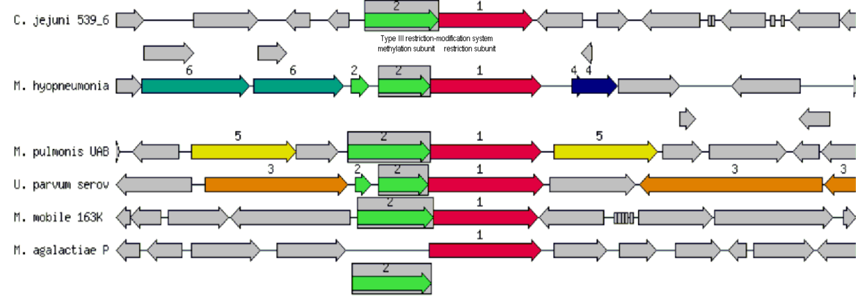
C. jejuni 539_6
Type III restriction-modification system
methylation subunit
restriction subunit
M. hyopneumonia
M. pulmonis UAB
U. parvum serov
M. mobile 163K
M. agalactiae P
1
2
3
4
5
6

Selected feature: bases 32501 misc_feature (/colour=255 175 175 /algorithm="alien_hunter" /score=24.212 /note="threshold: 14.773")

Entry: 40_677_677.gbk

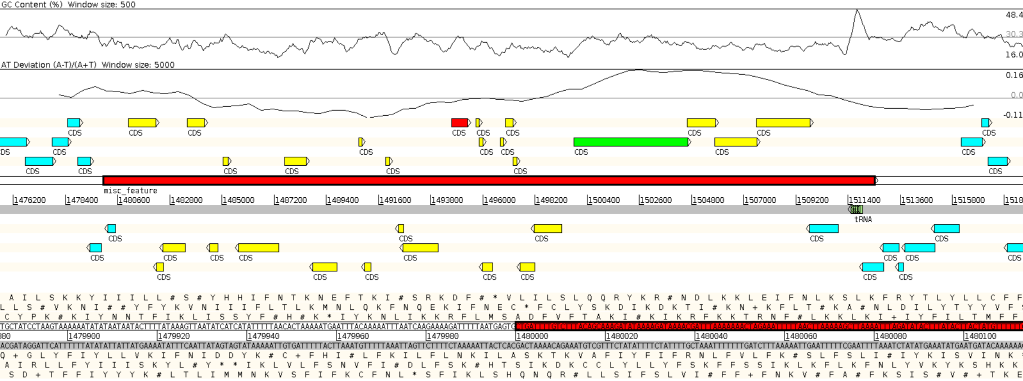


| | | | | |
|---|---|---|---|---|
| misc_feature | 1480000 | 1512500 | | |
| CDS | 1480181 | 1480486 | c | FIG00638667: hypothetical protein |
| CDS | 1481056 | 1482186 | | Integrase |
| CDS | 1482232 | 1482504 | c | hypothetical protein |
| CDS | 1482504 | 1483427 | c | hypothetical protein |
| CDS | 1483543 | 1484235 | | hypothetical protein |
| CDS | 1484475 | 1484795 | c | hypothetical protein |
| CDS | 1485042 | 1485236 | | IncF plasmid conjugative transfer protein TraG |
| CDS | 1485684 | 1487369 | c | hypothetical protein |
| CDS | 1487628 | 1488524 | | hypothetical protein |
| CDS | 1488832 | 1489839 | c | FIG00471674: hypothetical protein |
| CDS | 1490765 | 1490890 | | hypothetical protein |
| CDS | 1491010 | 1491252 | c | hypothetical protein |
| CDS | 1491918 | 1492046 | | hypothetical protein |
| CDS | 1492442 | 1492633 | c | conserved hypothetical protein |
| CDS | 1492626 | 1494083 | c | conserved hypothetical protein |
| CDS | 1494688 | 1495338 | | DNA/RNA non-specific endonuclease |
| CDS | 1495711 | 1495833 | | hypothetical protein |
| CDS | 1495859 | 1495972 | | hypothetical protein |
| CDS | 1495978 | 1496391 | c | FIG00469806: hypothetical protein |

Table S1. List of species included in our ε-proteobacterial database for orthology querying.

| **Species** | **Strain** | ***crp*†** | ***crp* locus tag/Accession** |
|---|---|---|---|
| *Campylobacter coli* | RM2228* | 1, 2 | 1) CCO0558<br>2) CCO0137 |
| *Campylobacter coli* | BIGS0002* | 1, 2 | |
| *Campylobacter coli* | BIGS0015* | 1, 2 | |
| *Campylobacter coli* | BIGS0019* | 1, 2 | |
| *Campylobacter coli* | BIGS0023* | 1, 2 | |
| *Campylobacter coli* | BIGS0024* | 1, 2 | |
| *Campylobacter coli* | BIGS0025* | 1 | |
| *Campylobacter coli* | BIGS0001** | 1, 2 | |
| *Campylobacter coli* | BIGS0010** | 1, 2 | |
| *Campylobacter coli* | BIGS0011** | 1, 2 | |
| *Campylobacter coli* | BIGS0012** | 1 | |
| *Campylobacter coli* | BIGS0003*** | 1,2 | |
| *Campylobacter coli* | BIGS0008*** | 1, 2 | |
| *Campylobacter coli* | 76339††*** | 1, 2 | 1) BN865_16100c<br>2) BN865_04590c |
| *Campylobacter jejuni* subsp. *doylei* | 269.97 | 1 | JJD26997_1473 |
| *Campylobacter jejuni* subsp. *doylei* | ATCC 49349 | 1 | T374_RS0103500 |
| *Campylobacter jejuni* subsp. *jejuni* | NCTC 11168 | 1 | Cj0466 (*nssR*) |
| *Campylobacter jejuni* subsp. *jejuni* | 81-176 | 1 | CJJ81176_0491 |
| *Campylobacter jejuni* subsp. *jejuni* | 81116 | 1 | C8J_0439 |
| *Campylobacter jejuni* subsp. *jejuni* | RM1221 | 1 | CJE0516 |
| *Campylobacter jejuni* subsp. *jejuni* | ATCC 33560 | 1 | cje135_01931 |
| *Campylobacter jejuni* subsp. *jejuni* | 00-2425 | 1 | N135_00514 |
| *Campylobacter jejuni* subsp. *jejuni* | 00-2426 | 1 | N564_00448 |
| *Campylobacter jejuni* subsp. *jejuni* | 00-2538 | 1 | N565_00499 |
| *Campylobacter jejuni* subsp. *jejuni* | 00-2544 | 1 | N755_00497 |
| *Campylobacter jejuni* subsp. *jejuni* | 10186 | 1 | N194_06230 |
| *Campylobacter jejuni* subsp. *jejuni* | 10227 | 1 | L084_05505 |
| *Campylobacter jejuni* subsp. *jejuni* | 1336 | 1 | C1336_000070034 |
| *Campylobacter jejuni* subsp. *jejuni* | 20176 | 1 | N195_09090 |
| *Campylobacter jejuni* subsp. *jejuni* | 255 | 1 | L034_06335 |
| *Campylobacter jejuni* subsp. *jejuni* | 260.94 | 1, 2 | 1) CJJ26094_0773<br>2) CJJ26094_0107 |
| *Campylobacter jejuni* subsp. *jejuni* | 30286 | 1 | N196_02170 |
| *Campylobacter jejuni* subsp. *jejuni* | 30318 | 1 | N212_08750 |
| *Campylobacter jejuni* subsp. *jejuni* | 32488 | 1 | M635_06665 |
| *Campylobacter jejuni* subsp. *jejuni* | 327 | 1 | CSU_1663 |
| *Campylobacter jejuni* subsp. *jejuni* | 4031 | 1 | BN867_04360 |
| *Campylobacter jejuni* subsp. *jejuni* | 414 | 1 | C414_000090034 |
| *Campylobacter jejuni* subsp. *jejuni* | 6399 | 1 | WP_002851096 |
| *Campylobacter jejuni* subsp. *jejuni* | 84-25 | 1 | CJJ8425_0490 |
| *Campylobacter jejuni* subsp. *jejuni* | BJ-CJD101 | 1 | J431_1419 |

| | | | |
|---|---|---|---|
| *Campylobacter jejuni* subsp. *jejuni* | CF93-6 | 1 | CJJCF936_0492 |
| *Campylobacter jejuni* subsp. *jejuni* | CG8421 | 1 | Cj8421_0467 |
| *Campylobacter jejuni* subsp. *jejuni* | CG8486 | 1 | Cj8486_0457 |
| *Campylobacter jejuni* subsp. *jejuni* | Cj2 | 1 | N215_03885 |
| *Campylobacter jejuni* subsp. *jejuni* | Cj3 | 1 | N216_06955 |
| *Campylobacter jejuni* subsp. *jejuni* | Cj5 | 1 | N213_08600 |
| *Campylobacter jejuni* subsp. *jejuni* | g113 | 1 | K696_RS0102290 |
| *Campylobacter jejuni* subsp. *jejuni* | H22082 | 1 | CJJH22082_RS0104305 |
| *Campylobacter jejuni* subsp. *jejuni* | HB93-13 | 1 | CJJHB9313_0476 |
| *Campylobacter jejuni* subsp. *jejuni* | HN-CJD07035 | 1, 2 | 1) J432_1615<br>2) J432_1025 |
| *Campylobacter jejuni* subsp. *jejuni* | IA3902 | 1 | CJSA_0436 |
| *Campylobacter jejuni* subsp. *jejuni* | ICDCCJ07001 | 1, 2 | 1) ICDCCJ07001_426<br>2) ICDCCJ07001_78 |
| *Campylobacter jejuni* subsp. *jejuni* | ICDCCJ07002 | 1, 2 | 1) H840_1673<br>2) H840_1029 |
| *Campylobacter jejuni* subsp. *jejuni* | JCM2013 | 1 | JCM2013_RS00825 |
| *Campylobacter jejuni* subsp. *jejuni* | K1 | 1, 2 | 1) N217_06470<br>2) N217_08280 |
| *Campylobacter jejuni* subsp. *jejuni* | LMG 9872 | 1, 2 | 1) Cje89_033337<br>2) Cje89_02922 |
| *Campylobacter jejuni* subsp. *jejuni* | M1 | 1 | CJM1_0448 |
| *Campylobacter jejuni* subsp. *jejuni* | OXC5341 | 1, 2 | |
| *Campylobacter jejuni* subsp. *jejuni* | OXC6332 | 1, 2 | |
| *Campylobacter jejuni* subsp. *jejuni* | OXC7095 | 1, 2 | |
| *Campylobacter jejuni* subsp. *jejuni* | OXC7345 | 1, 2 | |
| *Campylobacter jejuni* subsp. *jejuni* | OXC7358 | 1, 2 | |
| *Campylobacter jejuni* subsp. *jejuni* | OXC8290 | 1, 2 | |
| *Campylobacter jejuni* subsp. *jejuni* | P110B | 1 | CJJP110B_RS0104305 |
| *Campylobacter jejuni* subsp. *jejuni* | PT14 | 1 | A911_02275 |
| *Campylobacter jejuni* subsp. *jejuni* | R14 | 1 | H730_02910 |
| *Campylobacter jejuni* subsp. *jejuni* | S3 | 1 | CJS3_0458 |
| *Campylobacter fetus* subsp. *fetus* | 82-40 | 2, 3 | 2) CFF8240_0798<br>3) CFF8240_0428 |
| *Campylobacter fetus* subsp. *testudinum* | 03-427 | 2, 3 | 2) CFT03427_0437; CFT03427_1019<br>3) CFT03427_0790 |
| *Campylobacter fetus* subsp. *venerealis* | cfvi03/293 | 2, 3 | 2) CFVI03293_0425<br>3) CFVI03293_0951 |
| *Campylobacter fetus* subsp. *venerealis* | NCTC 10354 | 2, 3 | 2) CFV354_0489<br>3) CFV354_0872 |
| *Campylobacter lari* | RM2100 | 1, 2 | 1) Cla_0689<br>2) Cla_1155 |
| *Campylobacter concisus* | 13826 | 3 | CCC13826_1550 |
| *Campylobacter concisus* | ATCC 51561 | 3 | ATCC51561_651 |

| | | | |
|---|---|---|---|
| *Campylobacter concisus* | ATCC 51562 | 2, 3 | 2) ATCC51562_1399<br>3) ATCC51562_365 |
| *Campylobacter concisus* | UNSWCD | 3 | UNSWCD_209 |
| *Campylobacter curvus* | 525.92 | 3 | CCV52592_0436 |
| *Campylobacter curvus* | DSM6644 | 2, 3 | 2) C514_RS0107125<br>3) C514_RS0105485 |
| *Campylobacter upsaliensis* | DSM5365 | 2 | Q327_RS0102540 |
| *Campylobacter upsaliensis* | JV21 | 2 | HMPREF9400_0311 |
| *Campylobacter upsaliensis* | RM3195 | 2 | CUP0987 |
| *Arcobacter butzleri* | ED1 | 3 | ABED_0342; ABED_1940 |
| *Arcobacter butzleri* | JV22 | 3 | HMPREF9401_0465;<br>HMPREF9401_1276 |
| *Arcobacter butzleri* | RM4018 | 3 | Abu_0367 |
| *Helicobacter pylori* | 26695 | - | |
| *Helicobacter pylori* | HPAG1 | - | |
| *Helicobacter pylori* | J99 | - | |
| *Helicobacter pylori* | PeCan18 | - | |
| *Helicobacter acinonychis* | Sheeba | - | |
| *Helicobacter bilis* | ATCC 43879 | 1, 2 | 1) HRAG_01781<br>2) HRAG_00942 |
| *Helicobacter bilis* | ATCC 51630 | 1 | T368_RS0107190 |
| *Helicobacter bizzozeronii* | CIII1 | - | |
| *Helicobacter canadensis* | MIT98-5491 | 1, 2, 3 | 1) HCMG_01580<br>2) HCMG_01531<br>3) HCMG_00288 |
| *Helicobacter cetorum* | 00-7128 | - | |
| *Helicobacter cetorum* | MIT99-5656 | - | |
| *Helicobacter cinaedi* | ATCC BAA-847 | 1, 2, 3 | 1) HCBAA847_1282<br>2) HCBAA847_2282<br>3) HCBAA847_0807 |
| *Helicobacter cinaedi* | CCUG18818 | 1, 2, 3 | 1) HCCG_01607<br>2) HCCG_00684<br>3) HCCG_01135 |
| *Helicobacter cinaedi* | PAGU611 | 1, 2, 3 | 1) HCN_0709<br>2) HCN_2022<br>3) HCN_1161 |
| *Helicobacter hepaticus* | ATCC 51449 | 1, 2, 3 | 1) HH0166<br>2) HH1150<br>3) HH0012 |
| *Helicobacter mustelae* | 12198 | - | |
| *Helicobacter pullorum* | MIT98-5489 | 1, 2 | 1) HPMG_00981<br>2) HPMG_01899 |
| *Helicobacter winghamensis* | ATCC BAA-430 | 1, 2 | 1) HWAG_00610<br>2) HWAG_00492 |
| *Sulfurimonas denitrificans* | DSM1251 | 3 | Suden_1312/_1296/_1512 |

| | | | |
|---|---|---|---|
| *Wolinella succinogenes* | DSM1740 | 1, 3 | 1) WS1193<br>3) WS0913 (*dnr*)/WS1181 |

*Clade 1 *C. coli* (Sheppard *et al.* 2008, 2013)

**Clade 2 *C. coli* (Sheppard *et al.* 2008, 2013)

***Clade 3 *C. coli* (Sheppard *et al.* 2008, 2013)

†*crp*: 1) *Cj0466*-like; 2) ST677CC-like; 3) Other; -) absent

††Skarp-de Haan *et al.* 2014

****Strain belonging to the Oxfordshire sentinel surveillance scheme for human campylobacteriosis in Oxfordshire, UK. Deposited in the PubMLST database at pubmlst.org/campylobacter

Table S2. BlastP results of the ST-794 Type III res subunit against the Concise Microbial Protein Database

| **Organism** | **Accession** | **Length (aa)** | **Locus tag** | **E-value** | **Score** |
|---|---|---|---|---|---|
| *C. jejuni* strain 539 | CP010457 | 672 | RC45_08390 | - | - |
| *Helicobacter acinonychis* str. Sheeba | YP_664513 | 673 | Hac_0712 | 0 | 633.26 |
| *Mycoplasma conjunctivae* HRC/581 | YP_002960738 | 819 | MCJ_002240 | 2.77785e-30 | 129.835 |
| *Mycoplasma agalactiae* PG2 | YP_001256295 | 807 | MAG_1520 | 2.16259e-20 | 100.737 |
| *Aggregatibacter aphrophilus* NJ8700 | YP_003007037 | 762 | NT05HA_0537 | 5.02033e-12 | 74.4441 |
| *Parabacteroides distasonis* ATCC 8503 | YP_001303488 | 781 | BDI_2135 | 7.606e-12 | 74.0935 |
| *Streptobacillus moniliformis* DSM 12112 | YP_003306730 | 751 | Smon_1418 | 5.92191e-11 | 71.2889 |
| *Herminiimonas arsenicoxydans* | YP_001099019 | 840 | HEAR0698 | 1.51159e-06 | 57.2659 |
| *Eubacterium rectale* ATCC 33656 | YP_002937217 | 887 | EUBREC_1323 | 0.000273293 | 50.2544 |
| *Bradyrhizobium* sp. ORS 278 | YP_001203507 | 824 | BRADO1376 | 0.000763836 | 48.8521 |
| *Bacillus cereus* B4264 | YP_002365706 | 892 | BCB4264_A0973 | 0.00134965 | 48.151 |
| *Xanthomonas campestris* pv. *campestris* str. B100 | YP_001902467 | 882 | xccb100_1061 | 0.00564767 | 46.0475 |

Table S3. Accession numbers of the whole genome sequences of 55 *C. jejuni* strains isolated from human blood (n=31; strains 2-100) and faeces (n=24; strains 519-542).

| **Strain** | **Accession** |
|---|---|
| 2 | CP010472 |
| 8 | CP010465 |
| 10 | CP010478 |
| 12 | CP010487 |
| 13 | CP010461 |
| 14 | CP010502 |
| 16 | CP010481 |
| 24 | CP010467 |
| 26 | CP010470 |
| 32 | CP010496 |
| 33 | CP010497 |
| 34 | CP010484 |
| 36 | CP010479 |
| 39 | CP010503 |
| 40 | CP010510 |
| 41 | CP010482 |
| 47 | CP010459 |
| 52 | CP010505 |
| 58 | CP010460 |
| 59 | CP010494 |
| 61 | CP010511 |
| 62 | CP010493 |
| 64 | CP010468 |
| 73 | CP010475 |
| 78 | CP010507 |
| 85 | CP010504 |
| 86 | CP010485 |
| 92 | CP010488 |
| 94 | CP010464 |

| | |
|---|---|
| 95 | CP010486 |
| 100 | CP010462 |
| 519 | CP010471 |
| 520 | CP010501 |
| 521 | CP010476 |
| 522 | CP010463 |
| 523 | CP010508 |
| 524 | CP010480 |
| 525 | CP010469 |
| 526 | CP010477 |
| 527 | CP010506 |
| 528 | CP010500 |
| 529 | CP010491 |
| 530 | CP010489 |
| 531 | CP010492 |
| 532 | CP010490 |
| 533 | CP010458 |
| 534 | CP010473 |
| 535 | CP010483 |
| 536 | CP010474 |
| 537 | CP010498 |
| 538 | CP010495 |
| 539 | CP010457 |
| 540 | CP010509 |
| 541 | CP010466 |
| 542 | CP010499 |
